# Trait mindfulness differentiates the interest in healthy diet from orthorexia nervosa

**DOI:** 10.1007/s40519-020-00927-2

**Published:** 2020-05-22

**Authors:** J. Strahler

**Affiliations:** 1 Psychotherapy and Systems Neuroscience, Justus-Liebig-University Giessen, Otto-Behaghel-Str. 10H, 35394 Giessen, Germany; 2 Health Psychology and Applied Diagnostics, Bergische University Wuppertal, Wuppertal, Germany

**Keywords:** Healthy orthorexia, Orthorexia nervosa, Mindfulness, Gender

## Abstract

**Background:**

Obsessive healthy eating and its extreme form orthorexia nervosa are epidemiologically significant problems. Mindfulness, the focused, non-judgmental attention to and awareness of present events, may be an important psychological contributor to (orthorexic) eating habits.

**Methods:**

In this cross-sectional survey-based study, 314 women and 75 men (mean age_total sample_ = 27.17 years, SD = 10.64) provided data on mindfulness (Freiburg Mindfulness Inventory, presence and acceptance subscale) and orthorexic eating (Teruel Orthorexia Scale, healthy orthorexia and orthorexia nervosa subscale).

**Results:**

In this study, we found a positive relation between mindfulness and healthy orthorexia, the non-pathological interest in eating healthy. By contrast, orthorexia nervosa, the pathological obsession with healthy eating, was negatively associated with mindfulness. Gender differences appeared neglectable.

**Conclusion:**

Taken together, these results confirm previous research showing that mindfulness encourages eating healthy and may protect against eating-related pathologies. Result also support the notion that orthorexia has two dimensions, healthy and nervosa, which are differently related to psychological factors, herein mindfulness.

**Level of evidence:**

Level III, cohort study.

## Introduction

Despite the availability of guidelines on what makes a healthy and balanced diet, some individuals strive for a form of diet that is not necessarily directed towards meeting these guidelines, but is based on individualized perceptions of what constitutes healthy eating. In some cases, this focus on healthy eating can develop into an increasingly restricted diet and an obsessive relationship with healthy eating. As a form of disordered eating, this behavior has been termed orthorexia nervosa (ON) from the Greek “orthós” meaning correct and “órexis” signifying appetite [1]. Despite growing scientific research and increasing case numbers in therapeutic practice, diagnostic criteria have not yet been agreed upon [2]. From the various proposals, however, some key characteristics can be extracted: (1) an obsessive focus on eating healthy foods and a pathological preoccupation related to foods, (2) emotional consequences and self-punishment when not complying with dietary rules including compensatory behaviors, and (3) significant distress and/or psycho-social and physiological impairments [2]. While there seems to be agreement on the epidemiological relevance of orthorexic eating, the discussion on the phenomenon’s clinical relevance and whether ON should become an accepted diagnosis appears quite controversial [3].

In this regard, it has been proposed that health-conscious eating behaviors need to be distinguished from a pathological form of orthorexic eating [4]. Initial evidence shows that orthorexia consists of two components, the pathological dimension—Orthorexia nervosa (ON), and the non-pathological interest in eating healthy—Healthy Orthorexia [HeOr; 4]. Both dimensions are not only conceptually distinguishable but also may differently relate to mental health. While the ON dimension may relate to psychological distress and heightened negative affect, HeOr was associated with better well-being [4, 5]. Given the assumed clinical relevance of ON, it is, thus, important to identify psychological drivers of healthy or orthorexic eating. We propose mindfulness as one such moderating factor.

Mindfulness, described as the focused, non-judgmental attention to and awareness of present events [6] is a self-regulation process. As such, it is a relevant factor across various health (risk) behaviors, including (disordered) eating behaviors. Studies have shown that mindfulness encourages to make healthier food choices, to better control serving sizes, and to prefer low-calorie dense foods [7, 8]. Mindfulness has also been related to less impulsive eating [8]. On the other hand, disordered eating behavior may be inversely related to mindfulness [9]. Currently, it is unclear whether gender plays a role in the association between mindfulness and eating behaviors. However, some earlier studies found gender differences in dispositional mindfulness [8].

So far, no study has examined the link between mindfulness and orthorexic eating patterns. Conceptually relevant findings stem from studies in Yoga practitioners. Yoga is an activity that incorporates greater self-awareness and mindfulness, including lifestyle modifications and healthier eating. Currently, yoga practitioners are considered to be at-risk for developing ON [10]. As this study used a diagnostic tool with questionable psychometric properties [11], conclusions must be drawn carefully. On the contrary, other results indicate more yoga practice to be linked to healthier eating [12].

The aim of this study was to investigate the relationship between mindfulness and orthorexic eating behaviors, while considering the two dimensions of orthorexia, healthy and nervosa. From previous research, it was assumed that HeOr is positively linked to mindfulness; while, ON shows the opposite pattern. Given the earlier findings of gender differences in the prevalence of orthorexic eating attitudes [13] and trait mindfulness [8], analyses also examined whether gender influenced these associations.

## Methods

### Participants and procedure

This study adopted a cross-sectional survey-based research design and recruited a convenience sample. The study link (SosciSurvey.com) was distributed using mailing lists of two universities and through social media platforms (Facebook, Twitter, WhatsApp groups). Subjects participated voluntarily and provided informed consent by ticking a respective box at the first page of the online survey. This study was performed in line with the principles of the Declaration of Helsinki. Approval was granted by the Ethic Committee of the University of Wuppertal (reference: MS/BBL 190411).

The following inclusion criteria were employed: aged 18 years and older, and clearly assigning themselves to the male or female gender. In total, 389 complete data sets were available for analyses.

### Survey measures

The Teruel Orthorexia Scale [TOS; 4] was employed to investigate orthorexic eating behaviors. This 17-item scale allows for the assessment of the two proposed dimensions, healthy orthorexia (TOS-HeOr, 9 items) and orthorexia nervosa (TOS-ON, 8 items). Every item is rated on a Likert scale from 0 (completely disagree) to 3 (completely agree). The TOS was translated into German using forward translation by two independent translators. A bilingual expert checked for inadequate expressions in the translation and a pre-test with *n* = 5 students did not indicate ambiguous words or translations that need further adjustments. The two subscales showed good internal consistency in the present study (Cronbach’s *α*_TOS-HeOr_ = 0.84, *α*_TOS-ON_ = 0.89).

The German version of the revised 13-item short form of the Freiburg Mindfulness Inventory [FMI; 14] was used to measure mindfulness. This tool has been proposed to be suitable in generalized context where specific knowledge on mindfulness is not common. The FMI is intended to measure a general factor of mindfulness. However, following analysis favored a two-factor solution with a presence facet (five items), representing mindful presence, and an acceptance facet (eight items), representing self-acceptance or non-judgmental acceptance of experience. Responses are given on a 4-point Likert scale from 1 (rarely) to 4 (almost always). Internal consistencies of both sub-factors were average to good in the present study (Cronbach’s *α*_presence_ = 0.70, *α*_acceptance_ = 0.82).

Gathered demographic data included age, gender, weight and height to calculate body mass index (BMI), school education (primary, secondary, university), current occupation (student, other), and marital status (un-married, married, divorced).

### Statistical analyses

Group means were compared using *X*^2^ statistic and Student’s *t* test. Pearson’s correlation examined associations between study variables. Gender-specific correlations were compared using Fisher’s *Z*. Finally, hierarchical linear regressions examined if mindfulness (presence, acceptance subscale) explains variance in orthorexic eating (HeOr and ON, respectively) after accounting for the main effect of gender. The gender × FMI subscale two-way interactions were entered at step 3. Metric predictors were *z*-standardized before entering regression models. All assumptions of hierarchical multiple regression were met (no multicollinearity between predictors, *r* = 0.602, VIF < 10; Cook’s distance < 0.1; normally distributed residuals; scatterplots indicated homoscedasticity).

## Results

Data from 314 women and 75 men (mean age_total sample_ = 27.17 years, SD 10.64) were available for analyses. Table 1 shows descriptives of the sample as well as means and standard deviations of variables under study. Men as compared to women had a higher BMI (*t* = 3.65, *p* < 0.001, *g*_Hedges_ = − 0.469), were slightly older (*t* = 2.41, *p* = 0.018, *g*_Hedges_ = − 0.375), and reported higher acceptance scores (*t* = 2.25, *p* = 0.025, *g*_Hedges_ = − 0.288). None of the other variables differed (all *t* < 1.8, *p* > 0.08, all *X*^2^ < 1.30, *p* > 0.52).

**Table 1:** Means and standard deviations of sample descriptive characteristics and variables under study

Variable	Men (*n* = 75)	Women (*n* = 314)	Total (*n* = 389)
Age, *M* (SD)	30.36 (13.37)	26.41 (9.75)	27.17 (10.64)
BMI, *M* (SD)	24.70 (5.23)	22.58 (4.34)	22.99 (4.59)
Marital status			
Not married, *n* (%)	62 (82.7)	270 (86.0)	332 (85.3)
Married, *n* (%)	10 (13.3)	38 (12.1)	48 (12.3)
Divorced, *n* (%)	3 (4.0)	6 (1.9)	9 (2.3)
Educational level			
Primary, *n* (%)	0 (0.0)	2 (0.6)	2 (0.5)
Secondary, *n* (%)	3 (4.0)	12 (3.8)	15 (3.9)
University, *n* (%)	72 (96.0)	300 (95.5)	372 (95.6)
Occupation			
Student, *n* (%)	52 (69.3)	222 (70.7)	274 (70.4)
Other, *n* (%)	23 (30.7)	92 (29.3)	115 (29.6)
TOS-HeOr	10.91 (5.29)	11.84 (4.78)	11.66 (4.89)
TOS-ON	2.80 (3.23)	3.61 (4.50)	3.45 (4.29)
FMI-mindfulness	35.40 (6.44)	34.15 (6.05)	34.39 (6.14)
FMI-presence	13.63 (2.69)	13.61 (2.51)	13.61 (2.54)
FMI-acceptance	21.77 (4.39)	20.55 (4.20)	20.78 (4.26)

*M* mean, *SD* standard deviation, *BMI* Body mass index, *SSS* subjective social status, *TOS* Teruel Orthorexia Scale, *HeOr* healthy orthorexia, *ON* Orthorexia nervosa, *FMI* Freiburg Mindfulness Inventory

TOS-HeOr and TOS-ON were moderately correlated in the total sample (0.502, *p* < 0.001) and the genders were comparable (men *r* = 0.514, women *r* = 0.504, both *p* < 0.001, *Z* = 0.103, *p* = 0.459).

Mindfulness correlated positively with TOS-HeOr (*r* = 0.170, *p* < 0.001) and negatively with TOS-ON (*r* = − 0.172, *p* = 0.001). For the TOS-HeOr scale, the coefficient appeared higher in the female sample but the difference was not significant (TOS-HeOr *r*_men_ = 0.140, *p* = 0.230, *r*_women_ = 0.188, *p* = 0.001, *Z* = − 0.377, *p* = 0.353; TOS-ON *r*_men_ = − 0.186, *p* = 0.109, *r*_women_ = − 0.165, *p* = 0.003, *Z* = − 0.166, *p* = 0.434). The presence subscale was positively associated with TOS-HeOr (*r* = 0.246, *p* < 0.001) but was uncorrelated to TOS-ON (*r* = − 0.048, *p* = 0.346). Coefficients did not differ between men and women (TOS-HeOr *r*_men_ = 0.214, *p* = 0.065, *r*_women_ = 0.256, *p* < 0.001, *Z* = − 0.340, *p* = 0.367; TOS-ON *r*_men_ = − 0.035, *p* = 0.764, *r*_women_ = − 0.051, *p* = 0.371, *Z* = 0.123, *p* = 0.451). By contrast, analyses showed the acceptance subscale to negatively correlate with TOS-ON (*r* = − 0.219, *p* < 0.001) but no association with TOS-HeOr (*r* = 0.099, *p* = 0.052). Among women, however, the association with TOS-HeOr reached significance but this difference between the genders was not statistically significant (TOS-HeOr *r*_men_ = 0.075, *p* = 0.524, *r*_women_ = 0.118, *p* = 0.037, *Z* = − 0.332, *p* = 0.370; TOS-ON *r*_men_ = − 0.252, *p* = 0.029, *r*_women_ = − 0.208, *p* < 0.001, *Z* = − 0.355, *p* = 0.361).

The hierarchical multiple regression revealed that at step one, gender did not contributed to the regression model, *F* (1,387) = 2.20, *p* = 0.137, and accounted for 0.6% of the variation in TOS-HeOr. Introducing the mindfulness variables explained an additional 6.4% of variation and this change in *R*^2^ was significant, *F* (2,385) = 13.77, *p* < 0.001. Only the presence subscale appeared as a significant predictor (*b Z* = 1.41, *p* < 0.001; see Table 2 for the details). Finally, the addition of the two-way interactions did not add to the explained variance in TOS-HeOr (Δ*R*^2^ < 0.001, *F* (2,383) = 0.09, *p* = 0.916, total model *R*^2^ = 0.070). In regard to TOS-ON, hierarchical multiple regression showed no effect of gender, *F* (1,387) = 2.14, *p* = 0.145, accounting for 0.3% of the variation in TOS-ON. The mindfulness variables explained an additional 5.6% of variation and this change in *R*^2^ was significant, *F* (2,385) = 11.39, *p* < 0.001. Both subscales appeared as significant predictors, particularly acceptance (*b*_z presence_ = 0.56, *p* = 0.037; *b*_z acceptance_ = − 1.26, *p* < 0.001; see Table 2 for the details). Finally, the addition of the two-way interactions did not add to the explained variance in TOS-ON (Δ*R*^2^ < 0.001, *F* (2,383) = 0.04, *p* = 0.957, total model *R*^2^ = 0.061).

**Table 2:** Hierarchical multiple regression with mindfulness predicting orthorexic eating

	TOS-HeOr				TOS-ON			
	*b*	SE	*β*	*p*	*b*	SE	*β*	*p*
Step 1								
Gender	0.93	0.63	0.08	0.137	0.81	0.55	0.07	0.145
Step 2								
Gender	0.85	0.61	0.07	0.167	0.45	0.54	0.04	0.410
FMI-presence	1.41	0.30	0.29	< 0.001	0.56	0.27	0.13	0.037
FMI-acceptance	− 0.34	0.31	− 0.07	0.273	− 1.26	0.27	− 0.29	< 0.001
Step 3								
Gender	0.81	0.63	0.07	0.201	0.46	0.56	0.04	0.409
FMI-presence	1.40	0.68	0.29	0.039	0.64	0.60	0.15	0.283
FMI-acceptance	− 0.53	0.69	− 0.11	0.444	− 1.21	0.61	− 0.28	0.049
FMI-presence × gender	0.01	0.76	< 0.01	0.986	− 0.10	0.67	− 0.02	0.879
FMI-acceptance × gender	0.24	0.77	0.04	0.753	− 0.07	0.68	− 0.01	0.920

*b* regression coefficient, *SE* standard error, *β* standardized regression, *TOS* Teruel Orthorexia Scale, *HeOr* healthy orthorexia, *ON* Orthorexia nervosa, *FMI* Freiburg Mindfulness Inventory

## Discussion

Contributing to our understanding of psychological drivers of extreme healthy or orthorexic eating, this is the first study that examined the link between the two orthorexia dimensions, healthy and nervosa, and mindfulness. As expected, healthy orthorexia was positively linked to mindfulness; while, ON correlated negatively with the FMI sum score. Importantly, the two facets of mindfulness, presence and acceptance, showed varying correlational patterns. Self-reported higher presence scores were associated with the healthy dimension of orthorexic eating but uncorrelated with the nervosa dimension. Higher acceptance scores, on the other hand, did not correlate with the healthy dimension, but negatively with the nervosa dimension. Gender differences were small. Mindfulness correlated with healthy orthorexia more strongly in women, especially with the acceptance subscale. Overall, mindfulness accounted for only 5.6 and 6.4% of the variation in the TOS-ON and the TOS-HeOr dimensions, respectively, without any gender effects.

Current findings corroborate previous studies that relate mindfulness eating behaviors and risk for eating disorder pathology. Similar to this research, mindful presence was positively linked to the non-pathological interest in healthy eating [12]. Mindfulness may encourage healthier eating [7, 8] and may protect against the development of eating disorder pathology [9]. Accordingly, subjects reporting lower self-acceptance and non-judgmental acceptance of experience showed higher orthorexia nervosa. At first sight, these findings seem to contradict research on orthorexic eating in yoga practitioners. This group is considered to be at-risk for developing orthorexia nervosa [10]. However, it is important to note that the employed measurement tools, apart from restrictions in psychometric properties [11], do not allow measuring orthorexic eating but rather capture an interest in healthy eating.

Mindfulness is a complex construct and as such, the two aspects of mindfulness, which were considered in the present study, showed different association patterns. A higher focus on the present moment including bodily awareness (presence facet) was linked to more health-conscious eating while lower non-reactivity to the inner experience and an accepting attitude (acceptance facet) correlated with pathological orthorexia nervosa. These results thus underscore the significance both dimensions and how they differently relate to psychological factors. Whether mindfulness may also act as mediator of the link between orthorexia dimensions and mental health [4, 5] remains to be investigated.

Furthermore, results of the current study do not support the assumption that pathways of an association between mindfulness and orthorexic eating differ between men and women. The association between acceptance and healthy orthorexia was significant among women but not men. Regression analyses revealed no unique contribution of gender and no interaction with mindfulness to predict orthorexic behaviors. This suggests mindfulness to be a psychological contributor to orthorexia independent of gender. As there is almost no knowledge about gender differences in pathophysiological mechanisms and risk factors of orthorexic behaviors, these results remain preliminary.

This study has some limitations and, thus, the conclusions should only be considered preliminary evidence for the link between mindfulness and orthorexic eating. Present findings stem from correlational research not allowing for causal conclusions. Longitudinal and experimental studies will have to examine whether dispositional mindfulness or mindfulness-based interventions benefit health and protect against illness, including orthorexia nervosa. Second, current data were collected from mainly students (> 70%) and women were over-represented thereby biasing analyses and limiting interpretability and generalizability of results. Furthermore, the construct of orthorexia used in the present study was conceptualized with differentiable dimensions: healthy and nervosa. This two-factor solution may be conceptually valid but still needs to be verified in larger and ethnically diverse samples. Likewise, mindfulness was examined with a focus on the two components presence and acceptance, but did not include other aspects of mindfulness such as awareness and attention [6]. Internal consistencies of the used measures were in acceptable and good ranges.

In this study, we established that mindfulness differently predicted orthorexia nervosa and healthy orthorexia. Explained variance, however, was small, suggesting that the development of a pathological interest in eating healthy and orthorexia nervosa is multi-factorial. Socio-demographic factors, other psychological correlates, such as perfectionism or personality, and biological factors must also be considered [2]. The present study combines mindfulness and the interest in eating healthy, and is, thus, of importance for our understanding of developmental and maintenance factors of eating disorders as compared to mindful eating which could, thus, be a target for behavior change interventions.

### What is already known on this subject?

Obsessing over healthy eating may contribute to the development of orthorexia nervosa. Psychological drivers must be identified and we propose mindfulness as one such factor.

### What this study adds?

Higher mindfulness was linked to healthy orthorexia, the non-pathological interest in eating healthy. By contrast, orthorexia nervosa scores where higher in subjects with lower trait mindfulness. Mindfulness as a moderator of orthorexic eating may, thus, be a target for interventions.

## References

[1] Bratman S (1997) Health food junkie. Yoga J:42–50

[2] Cena H, Barthels F, Cuzzolaro M, Bratman S, Brytek-Matera A, Dunn T, Varga M, Missbach B, Donini LM (2019). Definition and diagnostic criteria for orthorexia nervosa: a narrative review of the literature. Eat Weight Disord.

[3] Strahler J, Stark R (2020). Perspective: classifying orthorexia nervosa as a new mental illness—much discussion little evidence. Adv Nutr.

[4] Barrada JR, Roncero M (2018). Bidimensional structure of the orthorexia: development and initial validation of a new instrument. An Psicol-Spain.

[5] Barthels F, Barrada JR, Roncero M (2019). Orthorexia nervosa and healthy orthorexia as new eating styles. PLoS ONE.

[6] Hayes AM, Feldman G (2004). Clarifying the construct of mindfulness in the context of emotion regulation and the process of change in therapy. Clin Psychol-Sci Pr.

[7] Beshara M, Hutchinson AD, Wilson C (2013). Does mindfulness matter? Everyday mindfulness, mindful eating and self-reported serving size of energy dense foods among a sample of South Australian adults. Appetite.

[8] Jordan CH, Wang W, Donatoni L, Meier BP (2014). Mindful eating: trait and state mindfulness predict healthier eating behavior. Pers Individ Differ.

[9] Moore M, Masuda A, Hill ML, Goodnight BL (2014). Body image flexibility moderates the association between disordered eating cognition and disordered eating behavior in a non-clinical sample of women: a cross-sectional investigation. Eat Behav.

[10] Valera JH, Ruiz PA, Valdespino BR, Visioli F (2014). Prevalence of orthorexia nervosa among ashtanga yoga practitioners: a pilot study. Eat Weight Disord.

[11] Valente M, Syurina EV, Donini LM (2019). Shedding light upon various tools to assess orthorexia nervosa: a critical literature review with a systematic search. Eat Weight Disord.

[12] Domingues RB, Carmo C (2019). Disordered eating behaviours and correlates in yoga practitioners: a systematic review. Eat Weight Disord.

[13] Strahler J (2019). Sex differences in orthorexic eating behaviors: a systematic review and meta-analytic integration. Nutrition.

[14] Sauer S, Strobl C, Walach H, Kohls N (2013). Rasch-Analyse des Freiburger Fragebogens zur Achtsamkeit. [Rasch analysis of the Freiburg Mindfulness Inventory.]. Diagnostica.

